# Supplementary material for: A rapid cell-free expression and screening platform for antibody discovery
Source: Nat Commun. 2023 Jul 3;14:3897. doi: 10.1038/s41467-023-38965-w (PMC10318062; doi:10.1038/s41467-023-38965-w)
Supplement: Supplementary file 1 — Supplementary Information [file 41467_2023_38965_MOESM1_ESM.pdf]

Supplementary Materials for:

Title: A rapid cell-free expression and screening platform for antibody discovery

Author list: Andrew C. Hunt[1,2], Bastian Vögeli[1,2], Ahmed O. Hassan[3], Laura Guerrero[1,2], Weston Kightlinger[1,2], Danielle J. Yoesep[1,2], Antje Krüger[1,2], Madison DeWinter[1,2,4], Michael S. Diamond[3,5,6,7], Ashty S. Karim[1,2], Michael C. Jewett[1,2,8,9,10]*

Affiliations:

[1]Department of Chemical and Biological Engineering, Northwestern University, Evanston, IL, 60208, USA
[2]Center for Synthetic Biology, Northwestern University, Evanston, IL, 60208, USA
[3]Department of Medicine, Washington University School of Medicine, St. Louis, MO, 63110, USA
[4]Medical Scientist Training Program, Northwestern University Feinberg School of Medicine, Chicago, IL 60611, USA
[5]Department of Molecular Microbiology, Washington University School of Medicine, St. Louis, MO, 63110, USA
[6]Department of Pathology & Immunology, Washington University School of Medicine, St. Louis, MO, 63110, USA
[7]Andrew M. and Jane M. Bursky Center for Human Immunology and Immunotherapy Programs, Washington University School of Medicine, St. Louis, MO, 63110, USA
[8]Chemistry of Life Processes Institute, Northwestern University, Evanston, IL, 60208, USA
[9]Robert H. Lurie Comprehensive Cancer Center, Northwestern University, Chicago, IL, 60611, USA
[10]Department of Bioengineering, Stanford University, Stanford, CA, 94305, USA

***Corresponding author:** Michael Jewett (m-jewett@northwestern.edu)

This PDF file includes:

Supplementary Figures 1 to 12
Supplementary Tables 1 to 2

Other Supplementary Materials for this manuscript include the following:

Supplementary Data 1 to 3 and Source Data included as separate files.

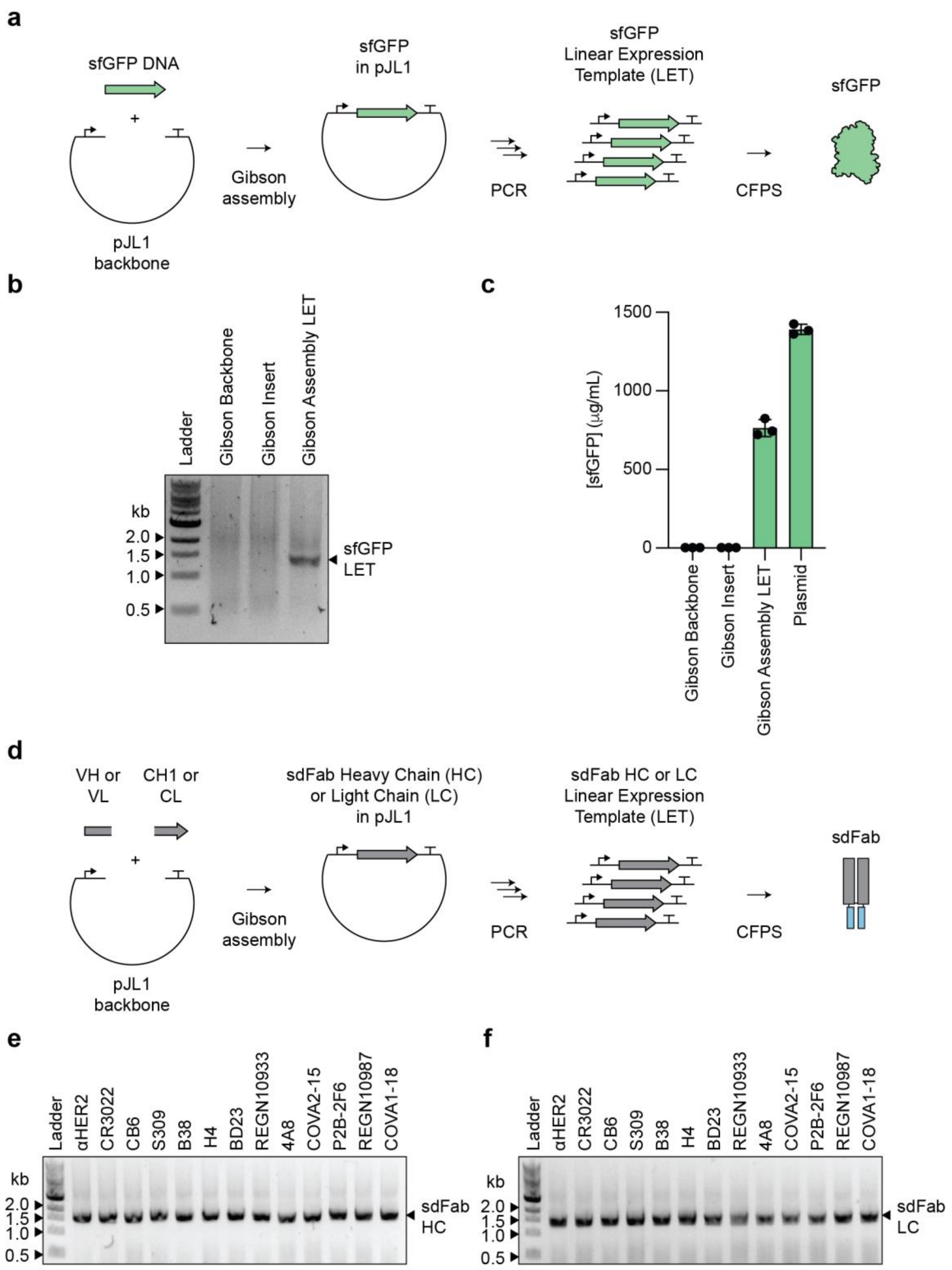

Supplementary Figure 1 | The cell-free DNA assembly and amplification workflow.

a, Schematic of the cell-free DNA assembly and amplification protocol for generating

sfGFP linear expression template for CFPS. **b**, Agarose gel of amplified LET PCR products of Gibson assembly reactions. Backbone only and insert only conditions included as negative controls representative of two independent experiments. Labeled band indicates assembly and amplification of the correct length PCR product. **c**, sfGFP yields in Origami^{TM} B(DE3) CFPS from cell-free assembled linear expression templates and purified plasmid (n = 3 independent CFPS replicates ± SEM). **d**, Schematic of the cell-free DNA assembly and amplification protocol for generating sdFab linear expression template for CFPS. **e**, Agarose gel of amplified sdFab heavy chain (HC) LET PCR products representative of two independent experiments. Labeled bands indicate assembly and amplification of the correct length PCR product. **f**, Agarose gel of amplified sdFab light chain (LC) LET PCR products representative of two independent experiments. Labeled bands indicate assembly and amplification of the correct length PCR product. Source data are provided as a Source Data.

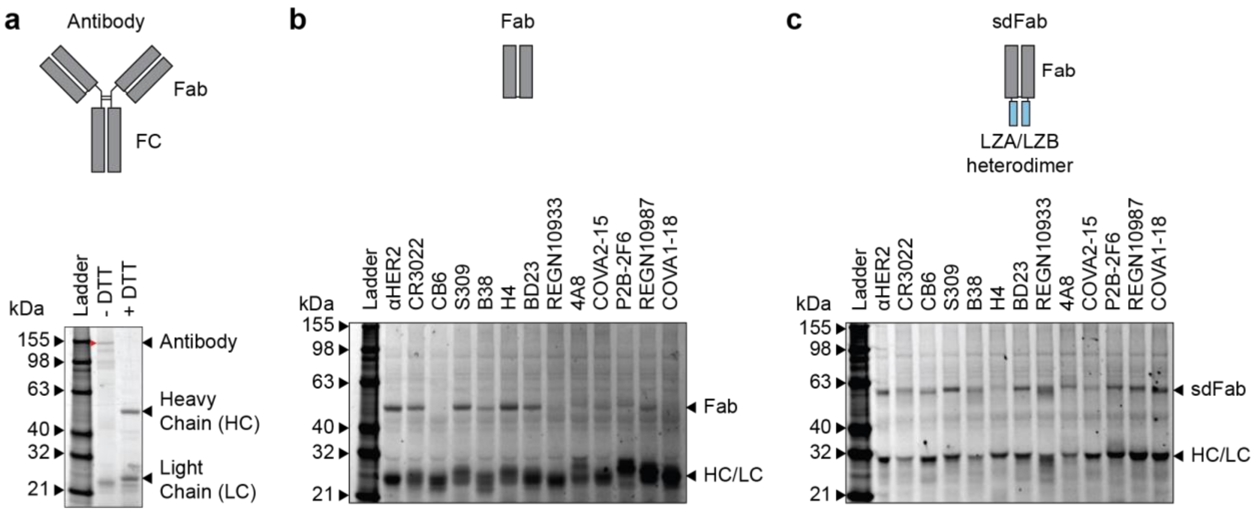

Supplementary Figure 2 | Development of an Origami™ B(DE3) CFPS system for the expression of antibodies and antibody fragments. **a-c**, SDS PAGE of antibodies and antibody fragments manufactured in CFPS representative of two independent experiments. Samples were fluorescently labeled with the FluoroTect™ reagent during protein synthesis. **a**, Expression and assembly of full-length Trastuzumab (αHER2). The full-length antibody (IgG, also highlighted by a red arrow), heavy chain (HC), and light chain (LC) are labeled. The full-length IgG band represents 14% of total antibody product measured by background subtracted densitometry. **b**, Expression and assembly of a panel of 13 Fabs (gel run in oxidizing conditions without DTT). **c**, Expression and assembly of a panel of 13 sdFabs (gel run in oxidizing conditions without DTT). Source data are provided as a Source Data.

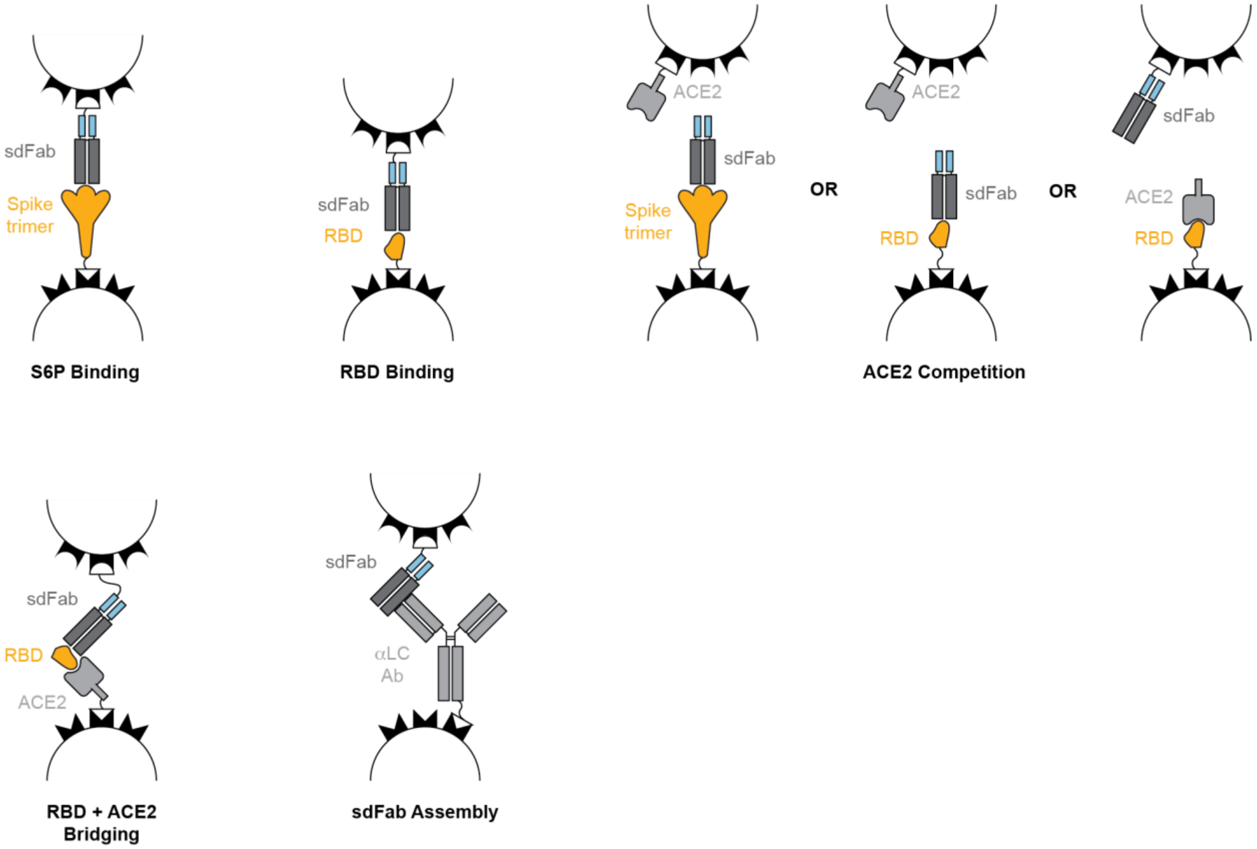

Supplementary Figure 3 | AlphaLISA measurement modalities used in this work.

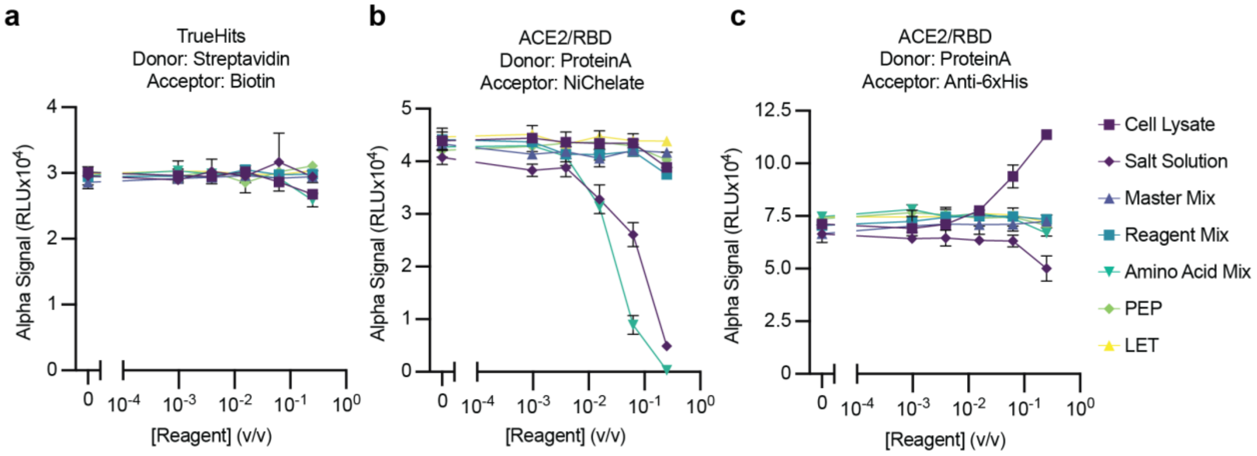

Supplementary Figure 4 | The impact of CFPS reagents on AlphaLISA signal. **a-c**, Evaluation of the effect of CFPS reagents on AlphaLISA (n = 3 independent replicates ± SEM). Concentrations are plotted as v/v fraction of the final concentration of the reagent in a CFPS reaction. Reagents were diluted in water at the concentration they normally reside at in CFPS. Reagents were tested in mixtures that were used to assemble CFPS reactions. The salt solution contains 8 mM magnesium glutamate, 10 mM ammonium glutamate, and 130 mM potassium glutamate. Master Mix contains 1.2 mM ATP, 0.85 mM GTP, 0.85 mM UTP, 0.85 mM CTP, 0.03 mg/mL folinic acid, and 0.17 mg/ml *E. coli* tRNA. Reagent Mix contains 0.4 mM NAD, 0.27 mM CoA, 4 mM oxalic acid, 1 mM putrescine, 1.5 mM spermidine, and 57 mM HEPES. Amino Acid Mix contains 2 mM of all 20 amino acids. PEP is 30 μM phosphoenolpyruvate. LET is 0.066 v/v fraction unpurified PCR mix containing the LET for sfGFP. **a**, Evaluation of the effect of CFPS reagents on AlphaLISA detection chemistry using the TrueHits kit. Biotin and Streptavidin labeled beads associate directly with one another and serve as a control for reagents impacting the AlphaLISA measurement chemistry. **b**, Evaluation of the effect of CFPS

reagents on AlphaLISA detection of the SARS-CoV-2 RBD and ACE2 interaction measured by the Protein A donor bead and Ni Chelate acceptor bead. **c**, Evaluation of the effect of CFPS reagents on AlphaLISA detection of the SARS-CoV-2 RBD and ACE2 interaction measured by the Protein A donor bead and anti-6xhis acceptor bead. Source data are provided as a Source Data.

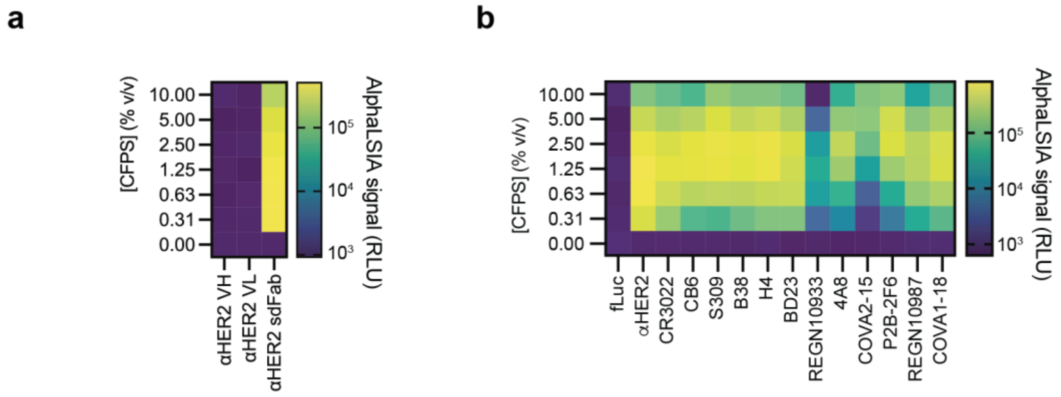

Supplementary Figure 5 | AlphaLISA for measuring assembly of CFPS derived sdFab. **a**, Assembly AlphaLISA measurement of a model anti-HER2 antibody fragment. Heavy and light chain were expressed either separately or together in a CFPS reaction. Only when both chains are co-expressed is assembly AlphaLISA signal observed. AlphaLISA signal is indicative of sdFab assembly, though the signal is subject to the hook effect[1], which can cause lower signal at higher concentrations. **b**, Assembly AlphaLISA measurement of firefly luciferase (fluc) as a non-antibody negative control and a panel of sdFabs. Source data are provided as a Source Data. **a-b**, AlphaLISA data are the mean of 3 replicates (n=3) derived from independent CFPS reactions.

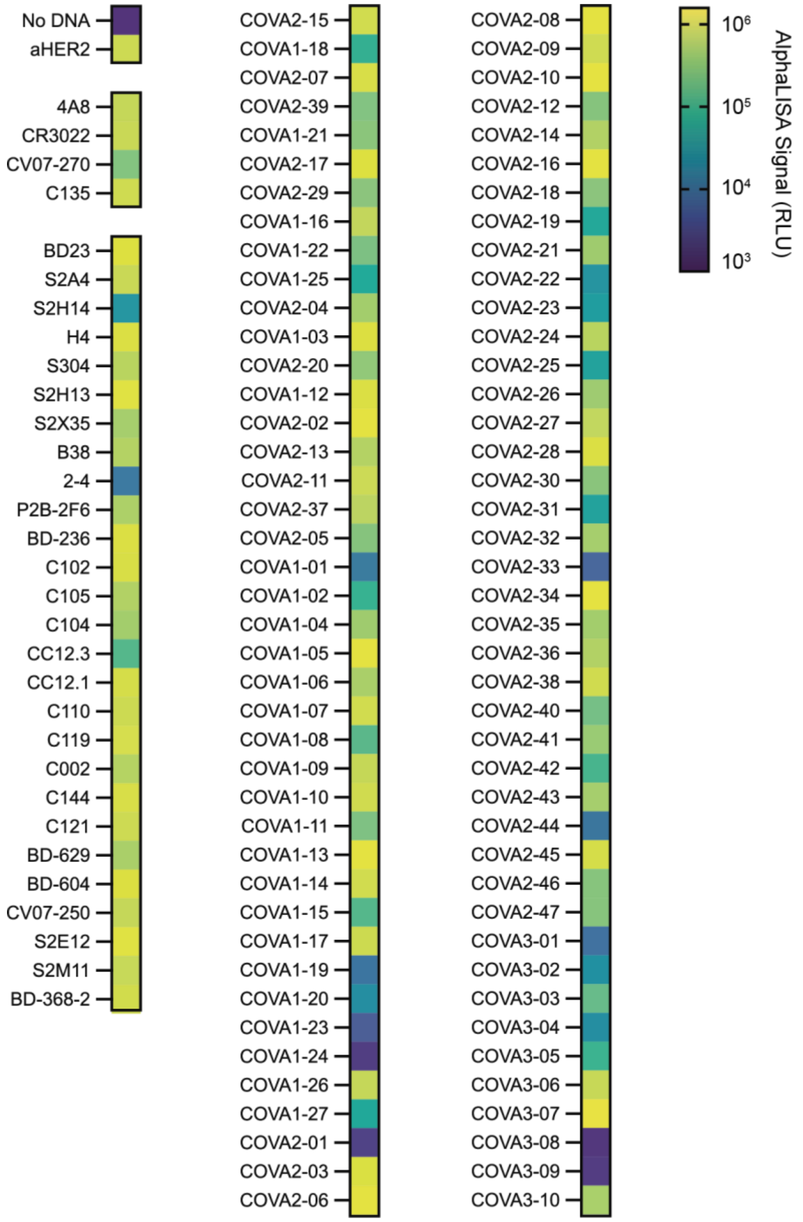

Supplementary Figure 6 | Assembly AlphaLISA measurements for the antibody sdFab fragments in Figure 2. Data are the mean of n = 3 independent CFPS replicates. Source data are provided as a Source Data.

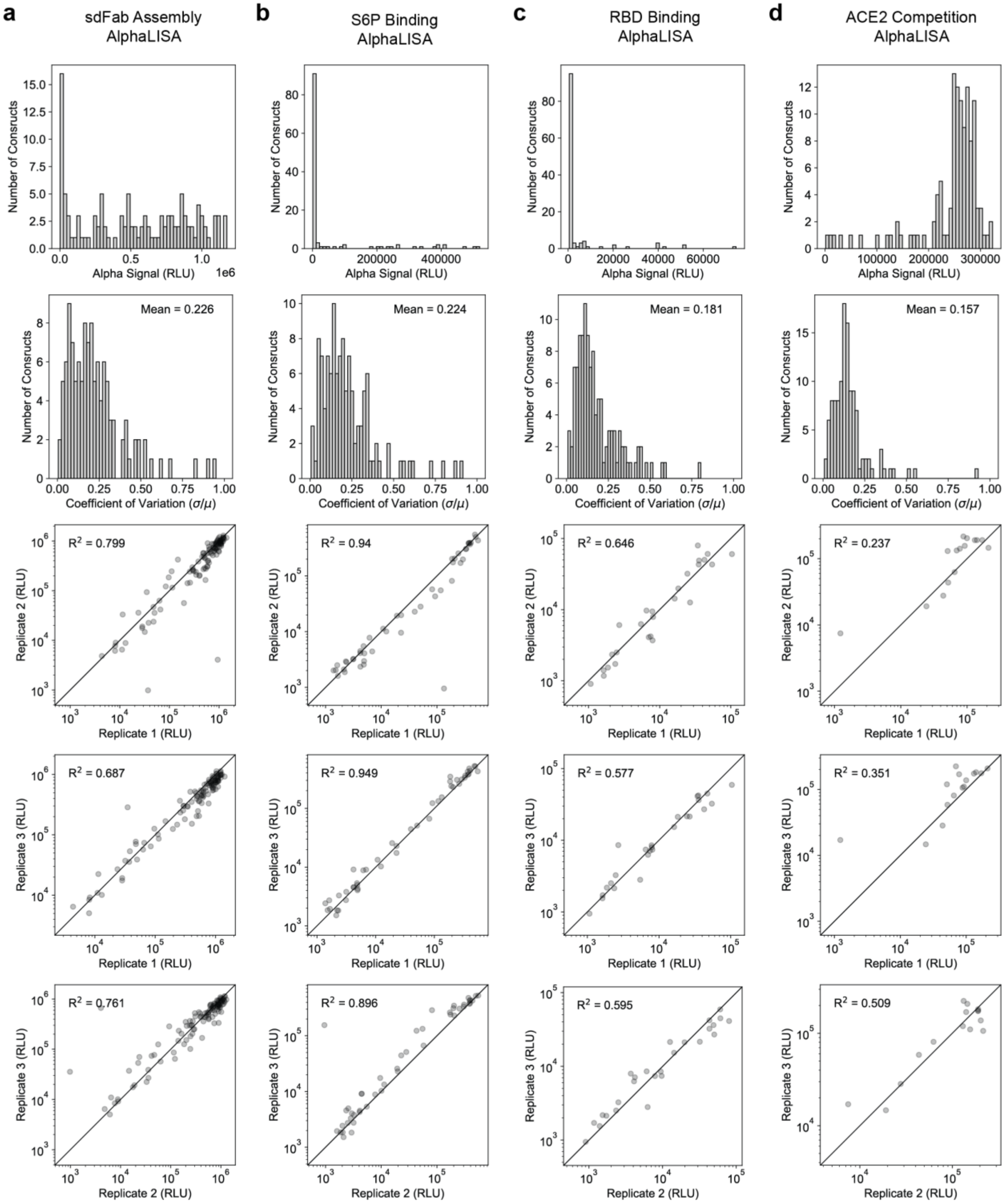

Supplementary Figure 7 | Analysis of variability in AlphaLISA replicates from the experiment constituting the data in Figure 2. **a-d**, From top to bottom: Histogram of

raw AlphaLISA values (mean of n = 3 independent CFPS replicates) to visualize the spread of the data. The number of constructs is the number of unique antibody fragments in a given histogram bin. A histogram of coefficient of variation (standard deviation divided by the mean) to visualize the typical error within a sample with the mean coefficient of variation displayed on the plot. The number of constructs is the number of unique antibody fragments in a given histogram bin. Parity plots of the three replicates were fit to the line y=x to visualize the consistency of replicates with the corresponding R^2 value is displayed on the chart. Only values found to be significantly different from the background are plotted ($p < 0.05$, two-sided t-test adjusted for multiple comparisons using FDR with a family-wise error rate of 5%) **a**, sdFab assembly AlphaLISA. **b**, SARS-CoV-2 S6P binding AlpahLISA. **c**, SARS-CoV-2 RBD binding AlphaLISA. **d**, sdFab competition with ACE2 for the SARS-CoV-2 RBD AlphaLISA. Source data are provided as a Source Data.

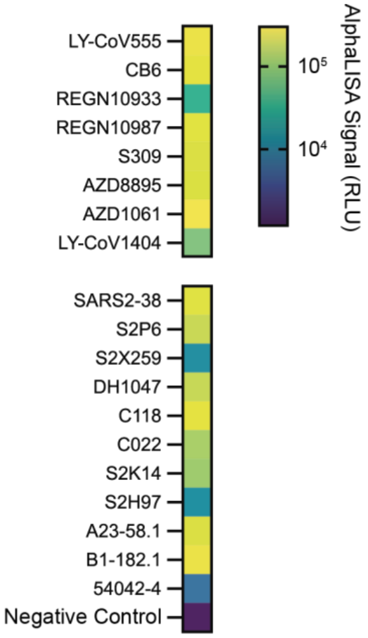

Supplementary Figure 8 | Assembly AlphaLISA measurements for the antibody fragments in Figure 3. Data are the mean of n = 3 independent CFPS replicates. Source data are provided as a Source Data.

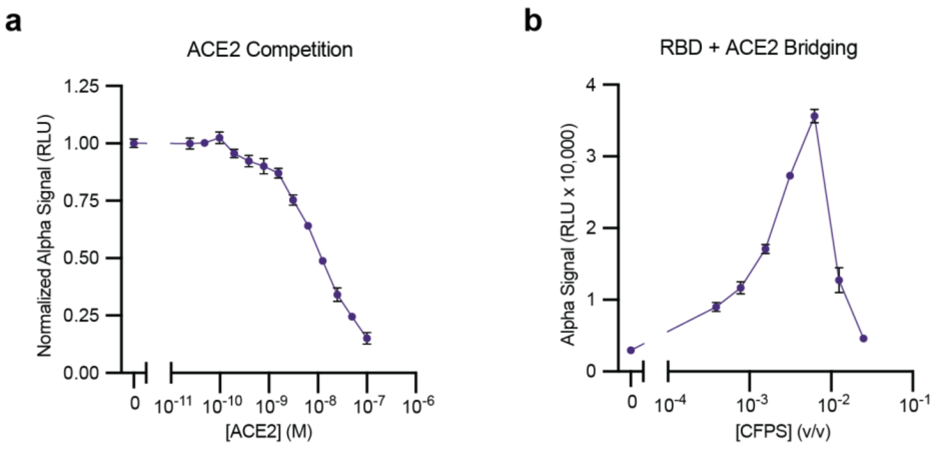

Supplementary Figure 9 | Analysis of ACE2 competition and RBD + ACE2 bridging AlphaLISA experiments for the S309 antibody fragments. **a**, ACE2 competition measured by titrating the concentration of ACE2. sdFabs in crude CFPS were diluted to a fixed concentration and combined with a fixed concentration of RBD and varying concentrations of ACE2 (mean of n = 3 independent replicates ± standard error of the mean). **b**, RBD + ACE2 bridging AlphaLISA (mean of n = 3 independent CFPS replicates ± standard error of the mean). In the ACE2 bridging experiment, the reduction in AlphaLISA signal at higher concentrations is likely due to the “hook effect”[1] where binding sites on the AlphaLISA beads become saturated and higher concentrations of antibody fragment begin to inhibit signal[1]. An absence of error bars inidicates error within the marker. Source data are provided as a Source Data.

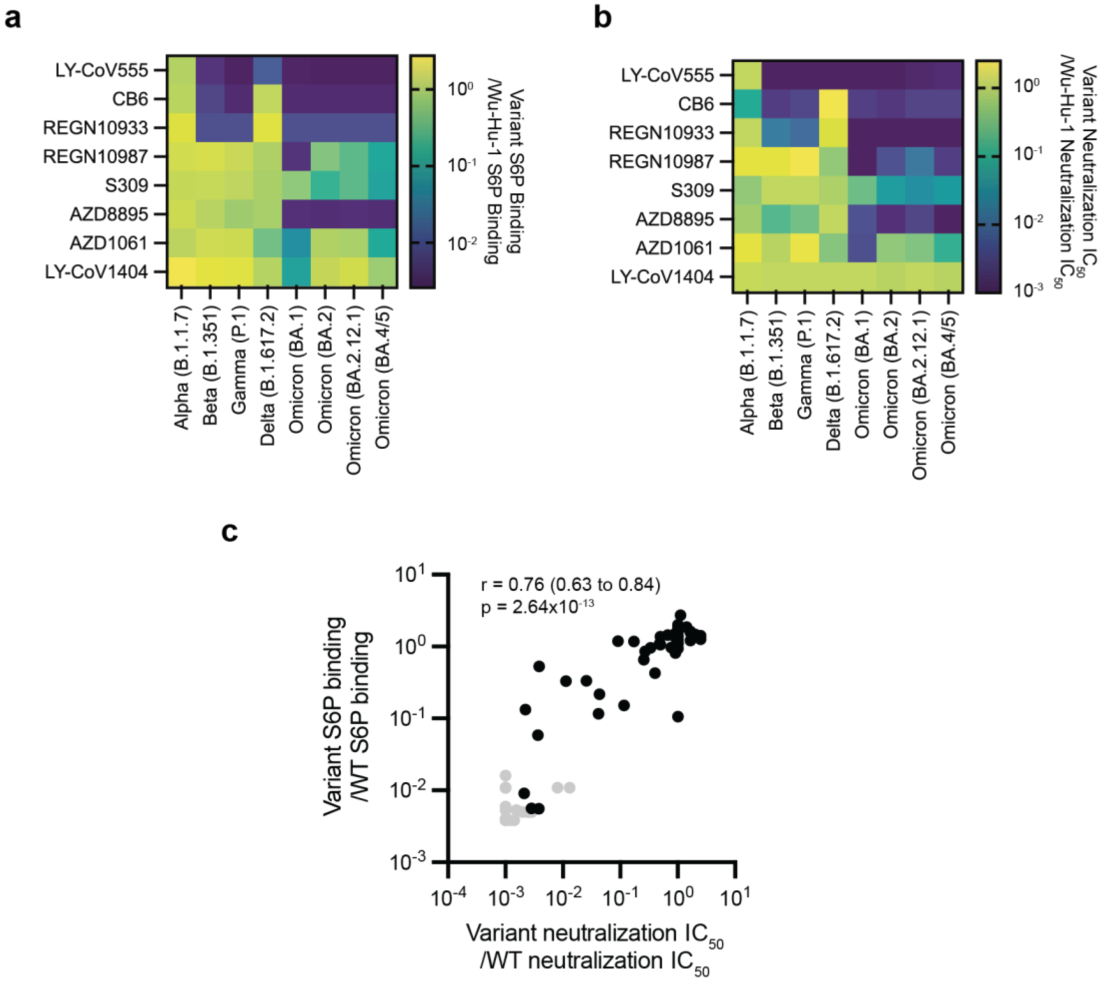

Supplementary Figure 10 | Comparison of AlphaLISA S6P binding data and neutralization data for historical emergency use authorized neutralizing antibodies against different current and historical SARS-CoV-2 variants of concern. **a**, Heatmap of background corrected SARS-CoV-2 S6P AlphaLISA data (mean of n = 3 independent CFPS replicates) normalized to Wu-Hu-1 S6P AlphaLISA data. **b**, Heatmap of SARS-CoV-2 variant neutralization IC_{50} values normalized to Wu-Hu-1 neutralization IC_{50} values. Data collected from the Standford Coronavirus Resistance Database[2]. **c**, Parity plot comparing variant AlphaLISA S6P binding data normalized to Wu-Hu-1 S6P binding data (mean of n = 3 independent CFPS replicates) to variant neutralization data

normalized to Wu-Hu-1 neutralization data. Data in grey are at the maximum dynamic range of either the AlphaLISA assay or the neutralization assay. Data in black are within the maximum dynamic range for both AlphaLISA and neutralization measurements. The dynamic range of the AlphaLISA measurement was determined by the signal for Wu-Hu-1 binding and the limit of detection (mean of background + 3 standard deviations). A Two-sided Pearson correlation coefficient was calculated using all data (within and outside of dynamic range) and the 95% confidence interval and two-tailed P value are displayed. Source data are provided as a Source Data.

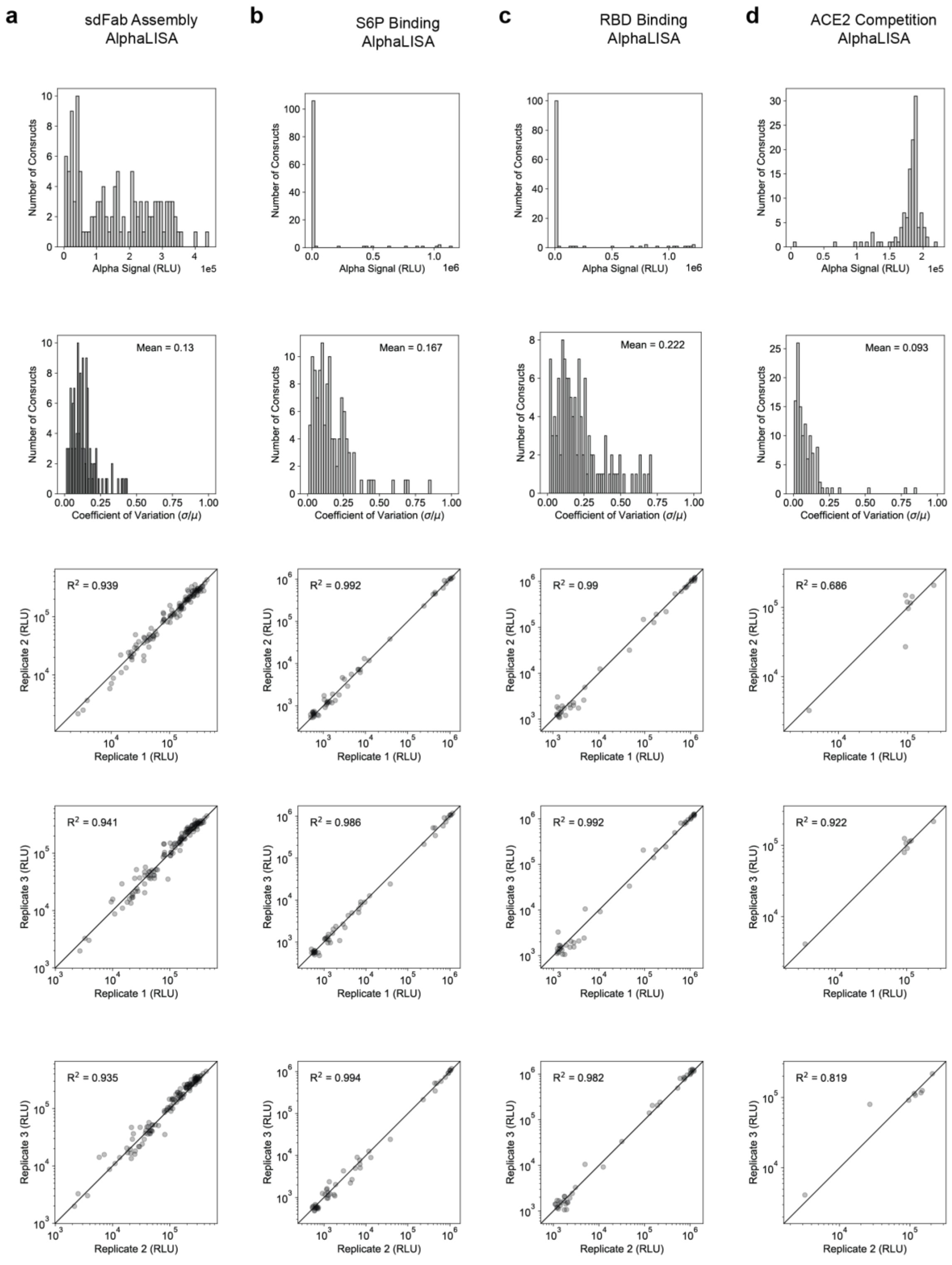

Supplementary Figure 11 | Analysis of variability in AlphaLISA replicates from the experiment constituting the data in Figure 3a and 4. a-d, From top to bottom:

Histogram of raw AlphaLISA values (mean of n = 3 independent CFPS replicates) to visualize the spread of the data. The number of constructs is the number of unique antibody fragments in a given histogram bin. A histogram of coefficient of variation (standard deviation divided by the mean) to visualize the typical error within a sample with the mean coefficient of variation displayed on the plot. The number of constructs is the number of unique antibody fragments in a given histogram bin. Parity plots of the three replicates were fit to the line y=x to visualize the consistency of replicates with the corresponding R^2 value is displayed on the chart. Only values found to be significantly different from the background are plotted ($p < 0.05$, two-sided t-test adjusted for multiple comparisons using FDR with a family-wise error rate of 5%) **a**, sdFab assembly AlphaLISA. **b**, SARS-CoV-2 S6P binding AlpahLISA. **c**, SARS-CoV-2 RBD binding AlphaLISA. **d**, sdFab competition with ACE2 for the SARS-CoV-2 RBD AlphaLISA. Source data are provided as a Source Data.

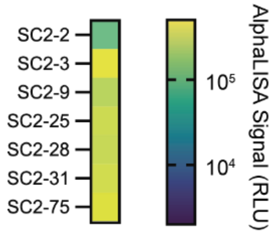

Supplementary Figure 12 | Assembly AlphaLISA measurements for the antibody sdFab fragments in Figure. Data are the mean of n = 3 independent CFPS replicates. Source data are provided as a Source Data.

Reference	DOI	Antibodies screened	Antibodies IC_{50} < 0.01 µg/mL	Antibodies IC_{50} < 0.25 µg/mL
Kreye *et al.*[3]	10.1016/j.cell.2020.09.049	598	5	40
Cao *et al.*[4]	10.1016/j.cell.2020.05.025	216	1	22
Wu *et al.*[5]	10.1126/science.abc2241	17	0	1
Liu *et al.*[6]	10.1038/s41586-020-2571-7	252	9	NR
Ju *et al.*[7]	10.1038/s41586-020-2380-z	206	0	3
Brouwer *et al.*[8]	10.1126/science.abc5902	84	2	9
Hansen *et al.*[9]	10.1126/science.abd0827	Thousands	6	NR
Robbani *et al.*[10]	10.1038/s41586-020-2456-9	94	9	43

Supplementary Table 1 | Summary of antibody screening studies designed to identify SARS-CoV-2 neutralizing antibodies. Studies were evaluated for their efficiency at identifying potently neutralizing antibodies. For each study, the total number of antibodies evaluated as well as the number of antibodies with a neutralization IC_{50} less than 0.01 µg/mL and 0.25 µg/mL were summarized. Either authentic- or pseudovirus neutralization IC_{50} was considered based on the breadth of the reported data. The 0.01 µg/mL cutoff was chosen because this value is approximately within an order of magnitude of the most potent reported neutralizing antibodies[6]. 0.25 µg/mL was chosen as a practical cutoff for moderately potent neutralizing antibodies[3]. For Hansen *et al.*[9] the neutralization potencies were reported in M and were converted to µg/mL assuming an antibody molecular weight of 150 kDa. For this analysis, only the studies containing antibodies expressed in this manuscript were used. Furthermore, only studies whose purpose was to identify neutralizing antibodies from a large set of candidates were considered. Studies that did not describe the results of their antibody discovery process in sufficient detail to collect the desired information were omitted. NR indicates not reported in sufficient detail to determine.

Experiment	Measurement	Donor Beads	Acceptor Beads	Component 1	Component 2	Component 3
Figure 1	ACE2 competition	Anti-Mouse IgG Alpha Donor beads (PerkinElmer, AS104)	Strep-Tactin AlphaLISA Acceptor beads (PerkinElmer, AL136)	Specified neutralizing antibody at variable concentration	SARS-CoV-2 RBD (Sino Biological, 40592-V02H) at 10 nM	Mouse FC tagged human ACE2 (Sino Biological, 10108-H05H) at 10 nM
Figure 2	Assembly	Anti-Rabbit IgG Alpha Donor beads (PerkinElmer, AS105)	Strep-Tactin AlphaLISA Acceptor beads (PerkinElmer, AL136)	CFPS w/ StrepII tagged sdFab at 0.025 v/v	Rabbit Anti-Human kappa light chain antibody (abcam, ab125919) OR Rabbit Anti-Human lambda light chain (abcam, ab124719) at 10 nM	
Figure 2	S6P binding	Strep-Tactin Alpha Donor beads (PerkinElmer, AS106)	Anti-6xHis AlphaLISA Acceptor beads (PerkinElmer, AL178)	CFPS w/ StrepII tagged sdFab at 0.025 v/v	Hisx6 tagged SARS-CoV-2 S6P (Acro Biosystems, SPN-C52H9) at 5 nM	
Figure 2	RBD binding	Anti-Human IgG Alpha Donor beads (PerkinElmer, AS114)	Strep-Tactin AlphaLISA Acceptor beads (PerkinElmer, AL136)	CFPS w/ StrepII tagged sdFab at 0.025 v/v	Human Fc-tagged SARS-CoV-2 RBD (Sino Biological, 40592-V02H) at 5 nM	
Figure 2	ACE2 competition	Anti-Human IgG Alpha Donor beads (PerkinElmer, AS114)	Anti-6xHis AlphaLISA Acceptor beads (PerkinElmer, AL178)	CFPS w/ StrepII tagged sdFab at 0.025 v/v	Biotinylated SARS-CoV-2 S6P (Acro Biosystems, SPN-C82E9) at 2 nM	Human FC-tagged human ACE2 (GenScript, Z03484) at 2 nM
Figure 3	Assembly	Anti-Rabbit IgG Alpha Donor beads (PerkinElmer, AS105)	Anti-FLAG AlphaLISA Acceptor Beads (PerkinElmer, AL112C)	CFPS w/ sFLAG tagged sdFab at 0.025 v/v	Rabbit Anti-Human kappa light chain antibody (abcam, ab125919) at 10 nM	
Figure 3	S6P binding	Anti-FLAG® Alpha Donor beads (PerkinElmer, AS103D)	Streptavidin AlphaLISA Acceptor beads (PerkinElmer, AL125C)	CFPS w/ sFLAG tagged sdFab at 0.025 v/v	Biotinylated SARS-CoV-2 S6P (Acro Biosystems, SPN-C82E9) at 50 nM	
Figure 3	RBD binding	Anti-FLAG® Alpha Donor beads (PerkinElmer, AS103D)	Streptavidin AlphaLISA Acceptor beads (PerkinElmer, AL125C)	CFPS w/ sFLAG tagged sdFab at 0.025 v/v	Biotinylated SARS-CoV-2 Spike RBD (Acro Biosystems, SPD-C82E9) at 50 nM	
Figure 3	ACE2 competition	Anti-Human IgG Alpha Donor beads (PerkinElmer, AS114)	Streptavidin AlphaLISA Acceptor beads (PerkinElmer, AL125C)	CFPS w/ sFLAG tagged sdFab at 0.025 v/v	Biotinylated SARS-CoV-2 Spike RBD (Acro Biosystems, SPD-C82E9) at 10 nM	Human FC-tagged human ACE2 (GenScript, Z03484) at 10 nM

Figure 3	VOC and other CoV	Anti-FLAG® Alpha Donor beads (PerkinElmer, AS103D)	Anti-6xHis AlphaLISA Acceptor beads (PerkinElmer, AL178)	CFPS w/ sFLAG tagged sdFab at 0.025 v/v	Specified Hisx6 tagged Spike protein at 10 nM	
Figure 4	Assembly	Anti-Rabbit IgG Alpha Donor beads (PerkinElmer, AS105)	Anti-FLAG AlphaLISA Acceptor Beads (PerkinElmer, AL112C)	CFPS w/ sFLAG tagged sdFab at 0.025 v/v	Rabbit Anti-Human kappa light chain antibody (abcam, ab125919) at 10 nM	
Figure 4	S6P binding	Anti-FLAG® Alpha Donor beads (PerkinElmer, AS103D)	Streptavidin AlphaLISA Acceptor beads (PerkinElmer, AL125C)	CFPS w/ sFLAG tagged sdFab at 0.025 v/v	Biotinylated SARS-CoV-2 S6P (Acro Biosystems, SPN-C82E9) at 50 nM	
Figure 4	RBD binding	Anti-FLAG® Alpha Donor beads (PerkinElmer, AS103D)	Streptavidin AlphaLISA Acceptor beads (PerkinElmer, AL125C)	CFPS w/ sFLAG tagged sdFab at 0.025 v/v	Biotinylated SARS-CoV-2 Spike RBD (Acro Biosystems, SPD-C82E9) at 50 nM	
Figure 4	ACE2 competition	Anti-Human IgG Alpha Donor beads (PerkinElmer, AS114)	Streptavidin AlphaLISA Acceptor beads (PerkinElmer, AL125C)	CFPS w/ sFLAG tagged sdFab at 0.025 v/v	Biotinylated SARS-CoV-2 Spike RBD (Acro Biosystems, SPD-C82E9) at 10 nM	Human FC-tagged human ACE2 (GenScript, Z03484) at 10 nM
Figure 4	VOC and other CoV	Anti-FLAG® Alpha Donor beads (PerkinElmer, AS103D)	Anti-6xHis AlphaLISA Acceptor beads (PerkinElmer, AL178)	CFPS w/ sFLAG tagged sdFab at 0.025 v/v	Specified Hisx6 tagged Spike protein at 10 nM	
Figure 4	Dose-dependent ACE2 competition	AlphaLISA Anti-FLAG donor (PerkinElmer, AS103D)	Streptavidin acceptor beads (PerkinElmer, AL125C)	CFPS w/ sFLAG tagged sdFab at 0.025 v/v	Biotinylated SARS-CoV-2 Spike RBD (Acro Biosystems, SPD-C82E9) at 10 nM	Human FC-tagged human ACE2 (GenScript, Z03484) at variable concentration
Figure 4	Dose-dependent RBD bridging	Anti-Human IgG donor beads (PerkinElmer, AS114D)	Anti-FLAG acceptor beads (PerkinElmer, AL112C)	CFPS w/ sFLAG tagged sdFab at variable concentration	Biotinylated SARS-CoV-2 Spike RBD (Acro Biosystems, SPD-C82E9) at 10 nM	Human FC-tagged human ACE2 (GenScript, Z03484) at 10 nM

Supplementary Table 2 | Summary of AlphaLISA experimental conditions and reagents used in this study. Listed concentrations are the concentration of the individual component in the final AlphaLISA reaction.

Supplementary References

1. Newton, P., Harrison, P. & Clulow, S. A novel method for determination of the affinity of protein: protein interactions in homogeneous assays. *J. Biomol. Screen.* **13**, 674–682 (2008).

2. Tzou, P. L., Tao, K., Pond, S. L. K. & Shafer, R. W. Coronavirus Resistance Database (CoV-RDB): SARS-CoV-2 susceptibility to monoclonal antibodies, convalescent plasma, and plasma from vaccinated persons. *PLoS One* **17**, e0261045 (2022).

3. Kreye, J. *et al.* A Therapeutic Non-self-reactive SARS-CoV-2 Antibody Protects from Lung Pathology in a COVID-19 Hamster Model. *Cell* **183**, 1058-1069.e19 (2020).

4. Cao, Y. *et al.* Potent neutralizing antibodies against SARS-CoV-2 identified by high-throughput single-cell sequencing of convalescent patients' B cells. *Cell* (2020) doi:10.1016/j.cell.2020.05.025.

5. Wu, Y. *et al.* A noncompeting pair of human neutralizing antibodies block COVID-19 virus binding to its receptor ACE2. *Science* **1278**, eabc2241 (2020).

6. Liu, L. *et al.* Potent neutralizing antibodies against multiple epitopes on SARS-CoV-2 spike. *Nature* **584**, 450–456 (2020).

7. Ju, B. *et al.* Human neutralizing antibodies elicited by SARS-CoV-2 infection. *Nature* **584**, 115–119 (2020).

8. Brouwer, P. J. M. *et al.* Potent neutralizing antibodies from COVID-19 patients define multiple targets of vulnerability. *Science* **369**, 643–650 (2020).

9. Hansen, J. *et al.* Studies in humanized mice and convalescent humans yield a SARS-CoV-2 antibody cocktail. *Science* **0827**, eabd0827 (2020).

10. Robbiani, D. F. *et al.* Convergent antibody responses to SARS-CoV-2 in convalescent individuals. *Nature* 2020.05.13.092619 (2020) doi:10.1038/s41586-020-2456-9.
