# Supplementary material for: A rapid cell-free expression and screening platform for antibody discovery
Source: Nat Commun. 2023 Jul 3;14:3897. doi: 10.1038/s41467-023-38965-w (PMC10318062; doi:10.1038/s41467-023-38965-w)
Supplement: Supplementary file 3 — Description of Additional Supplementary Files [file 41467_2023_38965_MOESM3_ESM.pdf]

Description of Additional Supplementary Files Document

Supplementary Data 1 | Reported parameters and expected behaviors for tested antibodies in Figure 2. Information about antibody target epitope, pseudo- or authentic virus neutralization IC50, and equilibrium dissociation constant from literature about the antibodies used in this study are summarized. Data are presented in three tabs, one for antibodies from diverse sources, one for the Brouwer et al. data set, and one for the EUA and bnAb data set. Data are in a separate .xlsx file.

Supplementary Data 2 | Variable heavy and light chain DNA and protein sequences of tested antibody fragments. Sequences are classified by their heavy (VH) or light chain (VL) as well as the light chain class (kappa or lambda). The variable domain protein sequence, the variable domain E. coli codon-optimized DNA sequence, and the ordered DNA sequence containing all additional (Gibson assembly homology, n-terminal expression tag, etc.) sequences are listed. Sequences are listed in a separate .csv file.

Supplementary Data 3 | Variable heavy and light chain cDNA sequences for antibodies identified in this manuscript. The variable domain protein sequence and cDNA sequence for each antibody are listed. Sequences are listed in a separate .csv file.
