# Supplementary material for: A rapid cell-free expression and screening platform for antibody discovery
Source: Nat Commun. 2023 Jul 3;14:3897. doi: 10.1038/s41467-023-38965-w (PMC10318062; doi:10.1038/s41467-023-38965-w)
Supplement: Supplementary file 7 — Reporting Summary [file 41467_2023_38965_MOESM7_ESM.pdf]

nature portfolio

Corresponding author(s): Michael Jewett

Last updated by author(s): Mar 14, 2023

# Reporting Summary

Nature Portfolio wishes to improve the reproducibility of the work that we publish. This form provides structure for consistency and transparency in reporting. For further information on Nature Portfolio policies, see our Editorial Policies and the Editorial Policy Checklist.

## Statistics

For all statistical analyses, confirm that the following items are present in the figure legend, table legend, main text, or Methods section.

| n/a | Confirmed | |
|---|---|---|
| ☐ | ☒ | The exact sample size (*n*) for each experimental group/condition, given as a discrete number and unit of measurement |
| ☐ | ☒ | A statement on whether measurements were taken from distinct samples or whether the same sample was measured repeatedly |
| ☐ | ☒ | The statistical test(s) used AND whether they are one- or two-sided<br>*Only common tests should be described solely by name; describe more complex techniques in the Methods section.* |
| ☐ | ☒ | A description of all covariates tested |
| ☐ | ☒ | A description of any assumptions or corrections, such as tests of normality and adjustment for multiple comparisons |
| ☐ | ☒ | A full description of the statistical parameters including central tendency (e.g. means) or other basic estimates (e.g. regression coefficient) AND variation (e.g. standard deviation) or associated estimates of uncertainty (e.g. confidence intervals) |
| ☐ | ☒ | For null hypothesis testing, the test statistic (e.g. *F*, *t*, *r*) with confidence intervals, effect sizes, degrees of freedom and *P* value noted<br>*Give P values as exact values whenever suitable.* |
| ☒ | ☐ | For Bayesian analysis, information on the choice of priors and Markov chain Monte Carlo settings |
| ☒ | ☐ | For hierarchical and complex designs, identification of the appropriate level for tests and full reporting of outcomes |
| ☐ | ☒ | Estimates of effect sizes (e.g. Cohen's *d*, Pearson's *r*), indicating how they were calculated |

*Our web collection on statistics for biologists contains articles on many of the points above.*

## Software and code

Policy information about availability of computer code

| | |
|---|---|
| Data collection | No specialized software was used for data collection. |
| Data analysis | Single-cell BCR sequences were analyzed with Cell Ranger v3.1.0. AlphaLISA data were analyzed with Prism 9.5.1. Images were processed using ImageJ2 v2.9.0/1.53t. Plate reader data were processed using Python 3.8.8. The Python code to analyze plate reader data was not central to the research and was not deposited. |

For manuscripts utilizing custom algorithms or software that are central to the research but not yet described in published literature, software must be made available to editors and reviewers. We strongly encourage code deposition in a community repository (e.g. GitHub). See the Nature Portfolio guidelines for submitting code & software for further information.

## Data

Policy information about availability of data

All manuscripts must include a data availability statement. This statement should provide the following information, where applicable:

- Accession codes, unique identifiers, or web links for publicly available datasets
- A description of any restrictions on data availability
- For clinical datasets or third party data, please ensure that the statement adheres to our policy

The data generated in this study are available in the Source Data. Protein and DNA sequences for all antibodies expressed in this work are available in Supplementary Data 2. Original cDNA sequences for antibodies derived from immunized mice originating from this work are available in Supplementary Data 3 and

have also been deposited to GenBank (Accession Numbers OQ570981 - OQ571099 for VH sequences and OQ571100 - OQ571218 for VL sequences). The raw sequencing data have been deposited to the Sequence Read Archive under the accession number PRJNA974195.

## Human research participants

Policy information about studies involving human research participants and Sex and Gender in Research.

| | |
|---|---|
| Reporting on sex and gender | NA |
| Population characteristics | NA |
| Recruitment | NA |
| Ethics oversight | NA |

Note that full information on the approval of the study protocol must also be provided in the manuscript.

# Field-specific reporting

Please select the one below that is the best fit for your research. If you are not sure, read the appropriate sections before making your selection.

☒ Life sciences ☐ Behavioural & social sciences ☐ Ecological, evolutionary & environmental sciences

For a reference copy of the document with all sections, see nature.com/documents/nr-reporting-summary-flat.pdf

# Life sciences study design

All studies must disclose on these points even when the disclosure is negative.

| | |
|---|---|
| Sample size | No statistical method was used to predetermine sample size. Previous experience with the measurement techniques and their dynamic ranges was used to determine sample sizes. |
| Data exclusions | One replicate of a control sample in the data related to Figure 4 was excluded from analysis due to a liquid handling error. The replicate is included in the Source Data file and annotated as excluded. |
| Replication | Large scale experiments (i.e. Figures 2 and 4) were not replicated in a separate experiment in their entirety, but the top conditions were independently replicated in separate experiments with matching results. Other experiments were generally replicated in a separate experiment at least once, with the results matching the published data. |
| Randomization | The experiments were not randomized because no animal studies or clinical trials were performed. |
| Blinding | The Investigators were not blinded to allocation during experiments and outcome assessment because no animal studies or clinical trials were performed. |

# Reporting for specific materials, systems and methods

We require information from authors about some types of materials, experimental systems and methods used in many studies. Here, indicate whether each material, system or method listed is relevant to your study. If you are not sure if a list item applies to your research, read the appropriate section before selecting a response.

### Materials & experimental systems

| n/a | Involved in the study | |
|---|---|---|
| ☐ | ☒ | Antibodies |
| ☒ | ☐ | Eukaryotic cell lines |
| ☒ | ☐ | Palaeontology and archaeology |
| ☐ | ☒ | Animals and other organisms |
| ☒ | ☐ | Clinical data |
| ☒ | ☐ | Dual use research of concern |

### Methods

| n/a | Involved in the study | |
|---|---|---|
| ☒ | ☐ | ChIP-seq |
| ☒ | ☐ | Flow cytometry |
| ☒ | ☐ | MRI-based neuroimaging |

## Antibodies

| | |
|---|---|
| Antibodies used | Antibody lot numbers were not recorded. All antibodies used were monoclonal. |

| | |
|---|---|
| Antibodies used | Manuscript Name: nAb1 \| Clone Name: AS35 \| Supplier: Acro Biosystems \| Catalog #: SAD-S35<br>Manuscript Name: : nAb2 \| Clone Name: NA \| Supplier: Sino Biological \| Catalog #: 40592-MM57<br>Manuscript Name: : nAb3 \| Clone Name: NA \| Supplier: Sino Biological \| Catalog #: 40591-MM43<br>Manuscript Name: : nAb4 \| Clone Name: NA \| Supplier: Sino Biological \| Catalog #: 40592-R001<br>Manuscript Name: : aK \| Clone Name: H19-5 \| Supplier: abcam \| Catalog #: ab125919<br>Manuscript Name: : aL \| Clone Name: EPR5367-62 \| Supplier: abcam \| Catalog #: ab124719<br>Manuscript Name: : anti-CD19 BV421 \| Clone Name: 6D5 \| Supplier: BioLegend \| Catalog #: 115537<br>Manuscript Name: : anti-CD4 FITC \| Clone Name: GK1.5 \| Supplier: BioLegend \| Catalog #: 100405<br>Manuscript Name: : anti-IgD-PE-Cy7 \| Clone Name: 11-26.c.2a \| Supplier: BioLegend \| Catalog #: 405719<br>Manuscript Name: : anti-mouse CD16/CD32 Fc block \| Clone Name: S17011E \| Supplier: BioLegend \| Catalog #: 156607 |
| Validation | No additional validation was performed beyond manufacturer validation.<br><br>Manuscript Name: nAb1 \| Supplier Validation: Antibody validated by ELISA and SPR.<br>Manuscript Name: : nAb2 \| Supplier Validation: Antibody validated by pseudovirus neutralization, flow cytometry, and immunofluorescence.<br>Manuscript Name: : nAb3 \| Supplier Validation: Antibody validated by ELISA, pseudovirus neutralization, flow cytometry, and immunofluorescence.<br>Manuscript Name: : nAb4 \| Supplier Validation: Antibody validated by ELISA, pseudovirus neutralization, and flow cytometry.<br>Manuscript Name: : aK \| Supplier Validation: Antibody validated by ELISA and western blot.<br>Manuscript Name: : aL \| Supplier Validation: Antibody validated by ELISA, western blot, and immunofluorescence.<br>Manuscript Name: : anti-CD19 BV421 \| Supplier Validation: Each lot of this antibody is quality control tested by immunofluorescent staining with flow cytometric analysis.<br>Manuscript Name: : anti-CD4 FITC \| Supplier Validation: Each lot of this antibody is quality control tested by immunofluorescent staining with flow cytometric analysis.<br>Manuscript Name: : anti-IgD-PE-Cy7 \| Supplier Validation: Each lot of this antibody is quality control tested by immunofluorescent staining with flow cytometric analysis.<br>Manuscript Name: : anti-mouse CD16/CD32 Fc block \| Supplier Validation: Each lot of this antibody is quality control tested by immunofluorescent staining with flow cytometric analysis. |

## Animals and other research organisms

Policy information about studies involving animals; ARRIVE guidelines recommended for reporting animal research, and Sex and Gender in Research

| | |
|---|---|
| Laboratory animals | Female C57BL/6 (Strain: 000664) were purchased from The Jackson Laboratory. Six-week-old animals were used for immunization. |
| Wild animals | The study did not involve wild animals. |
| Reporting on sex | Findings do not only apply to only one sex. |
| Field-collected samples | Study did not involve samples collected from the field. |
| Ethics oversight | Animal studies were carried out in accordance with the recommendations in the Guide for the Care and Use of Laboratory Animals of the National Institutes of Health. The protocols were approved by the Institutional Animal Care and Use Committee at the Washington University School of Medicine (Assurance number A3381-01). |

Note that full information on the approval of the study protocol must also be provided in the manuscript.
